# Central projections of nociceptive input originating from the low back and limb muscle in rats

**DOI:** 10.1038/s41598-025-86832-z

**Published:** 2025-01-20

**Authors:** Ulrich Hoheisel, Rolf-Detlef Treede, Siegfried Mense, Toru Taguchi

**Affiliations:** 1 Department of Neurophysiology, Mannheim Center for Translational Neurosciences, Ruprecht- Karls-University Heidelberg, 68167 Mannheim, Germany; 2 Department of Psychiatry and Psychotherapy, Central Institute for Mental Health, Ruprecht- Karls-University Heidelberg, 68167 Mannheim, Germany; 3 Department of Physical Therapy, Faculty of Rehabilitation, Niigata University of Health and Welfare, 1398 Shimami-cho, Kita-ku, Niigata, 950-3198 Japan; 4 Institute for Human Movement and Medical Sciences (IHMMS), Niigata University of Health and Welfare, 1398 Shimami-cho, Kita-ku, Niigata, 950-3198 Japan

**Keywords:** Neuroscience, Sensory processing, Somatosensory system

## Abstract

Since clinical features of chronic muscle pain originating from the low back and limbs are different (higher prevalence and broader/duller sensation of low back muscle pain than limb muscle pain), spinal and/or supraspinal projection of nociceptive information could differ between the two muscles. We tested this hypothesis using c-Fos immunohistochemistry combined with retrograde-labeling of dorsal horn (DH) neurons projecting to ventrolateral periaqueductal grey (vlPAG) or ventral posterolateral nucleus of the thalamus (VPL) by fluorogold (FG) injections into the vlPAG or VPL. C-Fos expression in the DH was induced by injecting 5% formalin into the multifidus (MF, low back) or gastrocnemius-soleus (GS, limb) muscle. A double-labeled DH neuron showing both c-Fos-immunoreactive nucleus and retrogradely transported FG in the cytoplasm was considered as a nociceptive projection neuron. Consistent with DH somatotopy for proximal vs. distal cutaneous inputs, DH neurons with MF input were located in the most lateral area of laminae I − II (segments Th12 − L5), while those with GS input were located in the middle area of laminae I − II (L3 − L5). DH neurons projecting to the vlPAG were located in superficial DH, while those projecting to VPL were located in deep DH. Supraspinal projection derived from more spinal segments for MF input than for GS input. These data suggest that nociceptive input from low back muscles is integrated more in craniocaudal direction than for limb muscles, and that these signals are then forwarded to both PAG and thalamus and contribute to the different nature of muscle pain arising from the low back and limbs.

**Supplementary Information:**

The online version contains supplementary material available at 10.1038/s41598-025-86832-z.

## Introduction

Chronic secondary musculoskeletal pain as a symptom of some diseases, especially with an origin in low back muscles, is the main cause of disability worldwide^1,2^. Generally, muscle pains in the low back and limbs have similar causes (e.g., excessive mechanical load, ischemia, and tissue pathology) and perceived qualities with dull sensation when compared to cutaneous pain^3^. However, there are some differences between the pains originating from the two muscles. For instance, muscle pain in the low back is much more common than that in the limbs, particularly when it is chronic^4^. It is perceived as dull and aching pain and is frequently radiating not only within the back, but also to remote areas such as hip and lower legs (i.e., referred pain)^5,6^. Compared to muscle pain in the limbs, low back muscle pain has a strong emotional-affective component^7^, and is promoted by psychosocial factors^8^, which are thought to be critical for the transition from acute to chronic pain^9^.

Another difference between the two muscles is the composition and function: low back muscles contain more than 60% type I fibers^10^ and fulfil mainly postural functions, whereas the vastus lateralis muscle (a typical limb muscle of the leg) contains fewer type I fibers^11^ and has locomotor functions. In addition, trunk muscles fulfilling postural functions are controlled by the medial motor systems, whereas limb muscles fulfilling locomotion function are controlled by the lateral motor systems in the ventral horn of the spinal cord^12,13^. However, it remains unknown whether the different composition and function of muscle fibers and different somatotopic representations in the spinal ventral horn could be the reason for the distinct pain characteristics. One possible explanation for the difference is that spinal and/or supraspinal processing of nociceptive input differs between the two muscles, particularly with regard to the signal intensity and somatotopic representation.

Neuroanatomical and electrophysiological experiments in our previous studies revealed that nociceptive input from low back muscles including the multifidus muscle (MF) projected to neurons in the spinal dorsal horn (DH), some of which were projecting to the ventrolateral periaqueductal grey (vlPAG)^14–17^. The vlPAG and ventral posterolateral nucleus of the thalamus (VPL) are known as supraspinal regions receiving input from muscle nociceptors^18–21^. To date, however, spinal nociceptive projections originating from the low back and limb muscles has not been systematically compared. Likewise, supraspinal nociceptive pathways via projection neurons in the DH receiving input from the two muscles have not been addressed so far.

Thus, in the current study, we tested the hypothesis that neuroanatomical projections of nociceptive input from the low back and limb muscles to the spinal cord and/or supraspinal centers could be different, using a double-labeling technique, namely, immunohistochemical staining of c-Fos-immunoreactive (-ir) nuclei in the spinal DH neurons activated by peripheral (muscle) nociceptive stimuli combined with retrograde tracing of the DH neurons projecting to supraspinal centers (i.e., vlPAG or VPL). The MF and GS muscle were chosen as a typical low back and limb muscle, respectively.

## Materials and methods

### Experimental animals

The experiments were carried out using forty male Sprague-Dawley rats (320–520 g), which were purchased from the Interfaculty Biomedical Facility of the Heidelberg University. Experimental procedures and the number of animals used in this study were approved by the local ethics authority responsible for animal experimentation (regional board Karlsruhe), and executed in accordance with the German law on the protection of animals, following the guidelines outlined in the European Council Directive, the ethical proposals of the International Association for the Study of Pain^22^, and the ARRIVE guidelines for reporting experiments involving animals^23^.

### Experimental groups

The following experimental groups were included:

*Fluorogold (FG) injection into the ****vlPAG***:

Group 1. Injection of 5% formalin into MF (low back pain group, *n* = 5 rats).

Group 2. Injection of 0.9% NaCl into MF (control for Group 1, *n* = 5 rats).

Group 3. Injection of 5% formalin into GS (limb pain group, *n* = 5 rats).

Group 4. Injection of 0.9% NaCl into GS (control for Group 3, *n* = 5 rats).

*FG injection into the ****VPL***:

Group 5. Injection of 5% formalin into MF (low back pain group, *n* = 5 rats).

Group 6. Injection of 0.9% NaCl into MF (control for Group 5, *n* = 5 rats).

Group 7. Injection of 5% formalin into GS (limb pain group, *n* = 5 rats).

Group 8. Injection of 0.9% NaCl into GS (control for Group 7, *n* = 5 rats).

All rats in the Group 1 − 8 received an injection of FG for retrograde tracing. The FG injections were made under deep anesthesia using stereotaxic techniques (see section “*Stereotaxic injection*”). Injections of 5% formalin as acute muscle nociceptive stimuli or 0.9% NaCl (isotonic saline) were made into two sites of either MF or GS (see section “*Nociceptive input caused by formalin injection*”). Three rats in each of the Group 1 − 4 were selected for quantitative analysis of c-Fos-ir nuclei in the DH to examine spinal projections of nociceptive input from the MF and GS.

### Stereotaxic injection of FG into the vlPAG and VPL

**Fig. 1:**
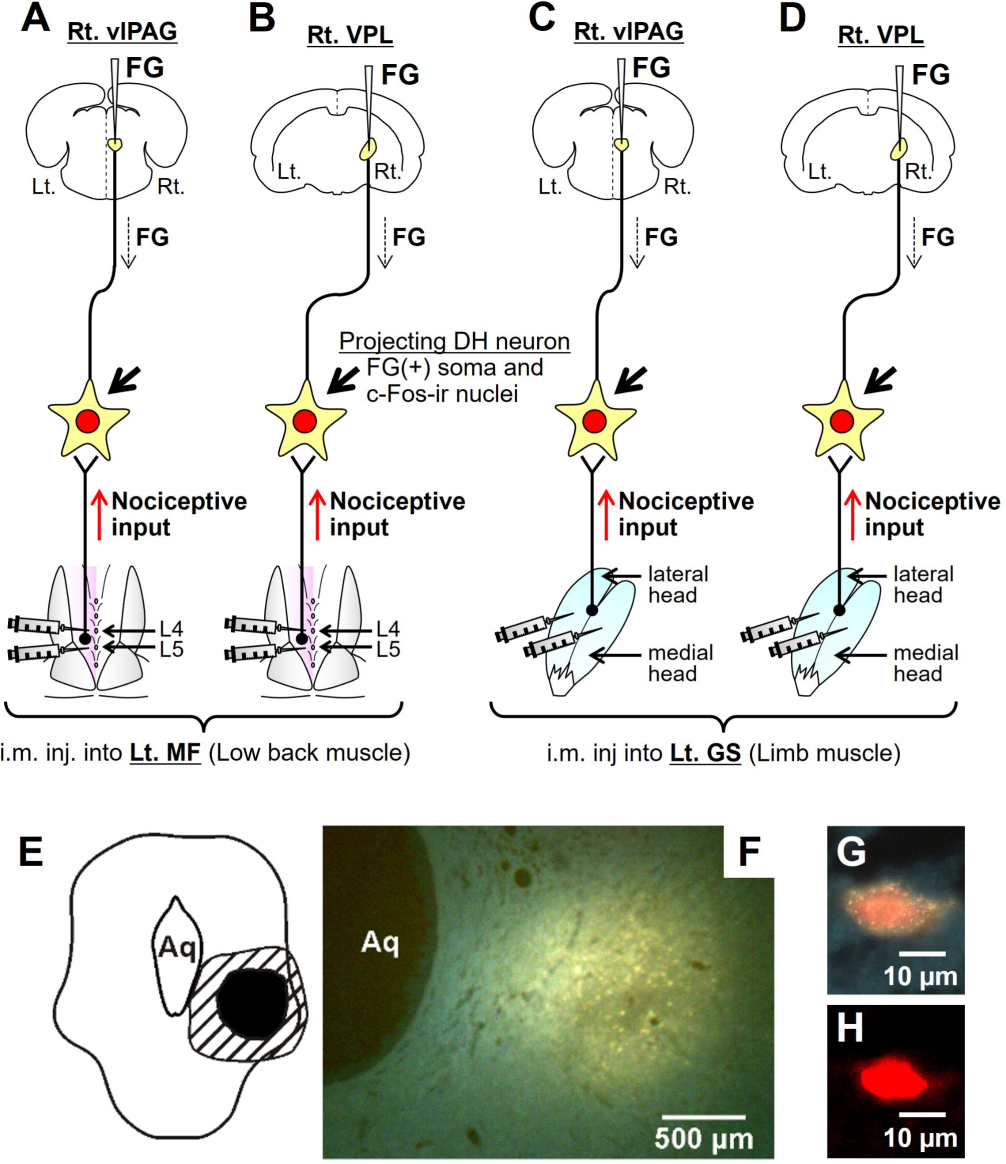
Experimental procedures for double-labeling of DH neurons. (**A−D**) Fluorogold (FG) injection was performed either into the right ventrolateral periaqueductal grey (vlPAG, **A** and **C**) or the right ventral posterolateral nucleus of the thalamus (VPL, **B** and **D**). Six days after FG injection into the brain region, intramuscular injection of 5% formalin or 0.9% saline was made either into the left multifidus muscle (MF) at the vertebral level L4 and L5 (10 µl each, **A** and **B**) or the medial and lateral head of the left gastrocnemius muscle (GS, 10 µl for each head, **C** and **D**). Double-labeled neurons that contained both the retrogradely transported FG in the cytoplasm (yellow) and c-Fos-ir in the nuclei (red) are spinal DH neurons receiving input elicited by muscle nociceptive stimuli (formalin) and projecting to the supraspinal centers. (**E**) Scheme of the fluorescent area in the right vlPAG where FG was injected. Aq: cerebral aqueduct, black area: strong fluorescence, hatched area: weak fluorescence. (**F**) The site of FG injection in **E**. (**G** and **H**) A double-labeled superficial DH neuron (at segment Th13), which was retrogradely stained from the vlPAG. **G**: FG labeling of a neuron, **H**: Same cell as in **G** with c-Fos-ir in its nucleus.

FG injections were made either into the vlPAG or VPL contralateral to the intramuscular injection of 5% formalin or 0.9% NaCl using stereotaxic techniques (Fig. 1). Stereotaxic injections were made according to the rat brain atlas^24^:

- vlPAG: AP, − 7.8 mm; L, 0.7 mm; V, 5.8 mm,
- VPL: AP, − 2.8 mm; L, 2.6 mm; V, 6.2 mm.

The rats were deeply anesthetized with a mixture of ketamine (100 mg/kg, i.p., Bela-Pharm GmbH & Co. KG, Germany) and xylazine (7.5 mg/kg, i.p., Bayer Vital GmbH, Germany). Fluorogold (FG or hydroxystilbamidine, 2%, diluted in distilled water; Molecular Probes, USA) was injected through fine glass micropipettes mounted in a stereotaxic apparatus (David Kopf Instruments, USA). The FG injections (0.1 µl) were made through a small craniotomy (diameter of 3–4 mm) in the dorsal skull. Following a survival period for 6 days, the rats received intramuscular injections of 5% formalin or 0.9% NaCl into the MF or GS (Fig. 1).

To see if the FG injections were restricted to the vlPAG and VPL, the mesencephalon and thalamus was sectioned and the spatial extent of fluorescence at the injection site was confirmed in three rats for each injection. The fluorescence was observed within the PAG and VPL with little diffusion to the surrounding area (Fig. 1).

Spinal segments Th12 − L6 were analyzed for double-labeled DH neurons that contained both the retrogradely transported FG in their cytoplasm and c-Fos-ir in their nuclei (Fig. 1). These two conditions (i.e., double-labeling with FG and c-Fos-ir) ensured that the neurons had connections to the vlPAG or VPL and received nociceptive input from the MF or GS muscle, respectively. In other words, double-labeled cells were considered as nociceptive neurons with muscle input and projection either to the vlPAG or VPL.

### Nociceptive input induced by formalin injection

This experiment was based on the fact that neuronal activation induced by acute nociceptive input from muscles was effective for inducing the expression of immediate early gene *c-fos* and its protein product c-Fos in the nuclei of spinal DH neurons^25–27^, and that injecting formalin into the muscle is one of the effective stimuli for the c-Fos induction^15^. 5% formalin solution was injected either into the left MF at the vertebral level L4 and L5 (10 µl each, Fig. 1A and B) or into the medial and lateral head (10 µl each, Fig. 1C and D) of the left GS. Injections of isotonic saline (0.9% NaCl) instead of formalin served as control. All injections were made with a 100 µl microsyringe through the skin under brief anesthesia with isoflurane (Baxter Germany, GmbH).

### C-Fos staining

Two hours after injection of formalin or saline, rats were euthanized using transcardial perfusion with 4% paraformaldehyde in 0.1 M phosphate buffered saline (PBS) followed by PBS containing 10% sucrose. After laminectomy, the segments Th12 − L6 were identified by exposing the dorsal root ganglia with their dorsal roots. Each segment was removed separately by cutting the cord at the cranial and caudal boundaries of their dorsal root entry zones. The segments were passed through PBS containing 30% sucrose for cryoprotection and snap frozen. Using a cryostat, serial cross sections were made from the spinal segments at a thickness of 20 μm. The procedure for visualizing nuclear c-Fos-ir in the spinal sections included the following steps:

- 10% normal goat serum and 0.1% Triton-X in PBS for 1 h at room temperature.
- Primary antibody to c-Fos (rabbit, 1:10,000, Merck Biosciences GmbH) for 24 h at room temperature.
- Biotinylated secondary antibody (anti-rabbit IgG, goat, 1:200, Vector Laboratories) for 2 h at room temperature.
- Fluorescent streptavidin-Cy3.5 (1:2,000, Biotrend Chemikalien GmbH) for 2 h at room temperature.
- Embedding in a mounting medium (Sakura Finetek Europe).

We verified that no immunoreactive signal was found when we omitted the primary or secondary antibody, immunostaining was always restricted to the nucleus of DH neurons, and that no staining was found in the cytoplasm.

### Data analysis

**Fig. 2:**
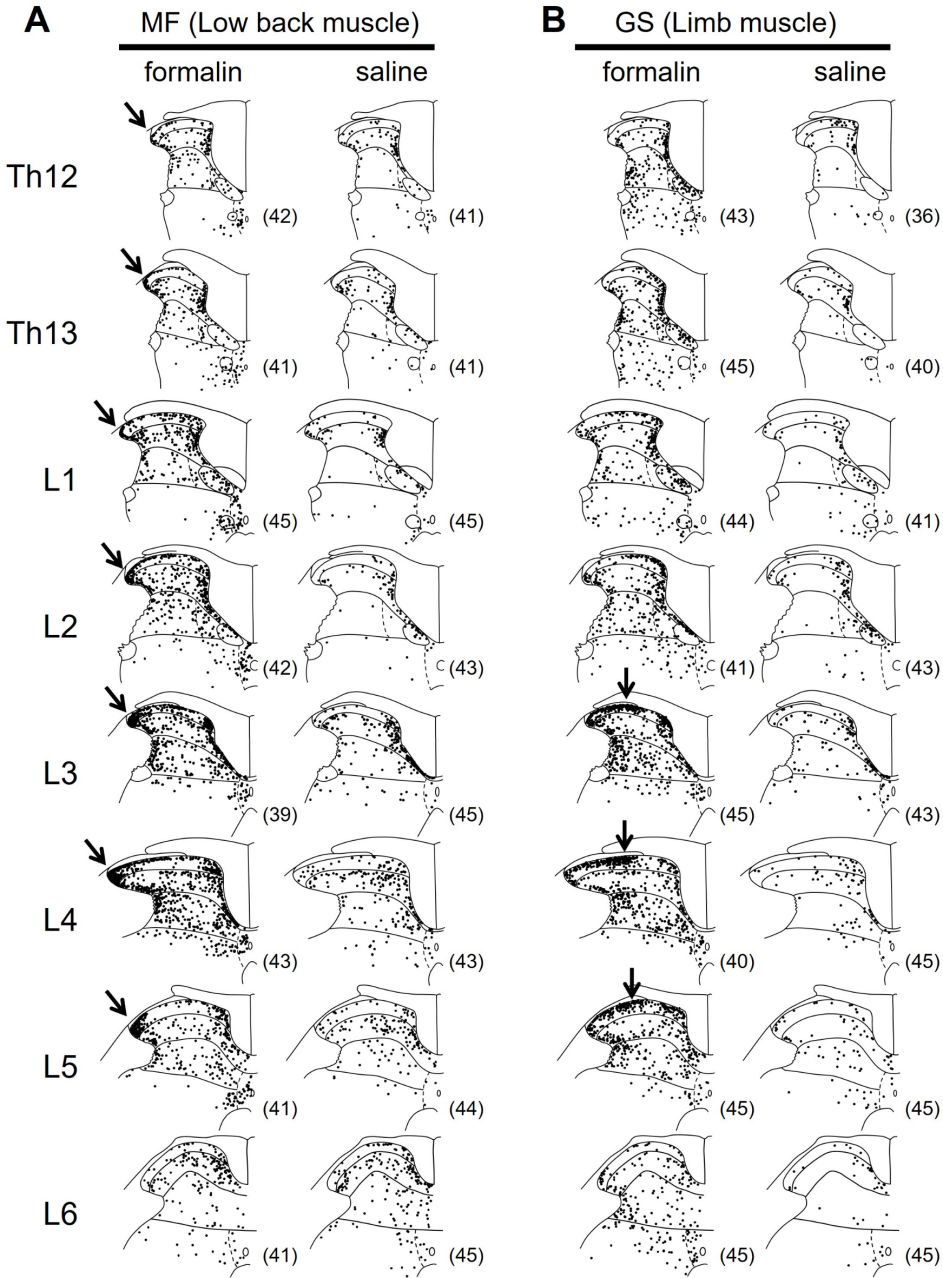
Somatotopic representation of c-Fos-ir nuclei in the DH after formalin or saline injection into the MF or GS muscle. (**A**) Distribution of c-Fos-ir nuclei at the spinal segments Th12–L6 in response to formalin or saline injection into the left MF (low back muscle). (**B**) That into the left GS (limb muscle). Each dot indicates a c-Fos-ir nucleus. In each panel, 36–45 sections from 3 rats were superimposed so that somatotopic representation of nociceptive input to the DH could be better visualized. The number of sections superimposed is shown in parenthesis. Note the somatotopically different expression patterns of c-Fos-ir nuclei (indicated by arrows): Dense labeling in the most lateral area of the superficial DH at segments Th12–L5 after formalin injection into the MF and that in the middle area of the superficial DH at segments L3–L5 after formalin injection into the GS.

**Fig. 3:**
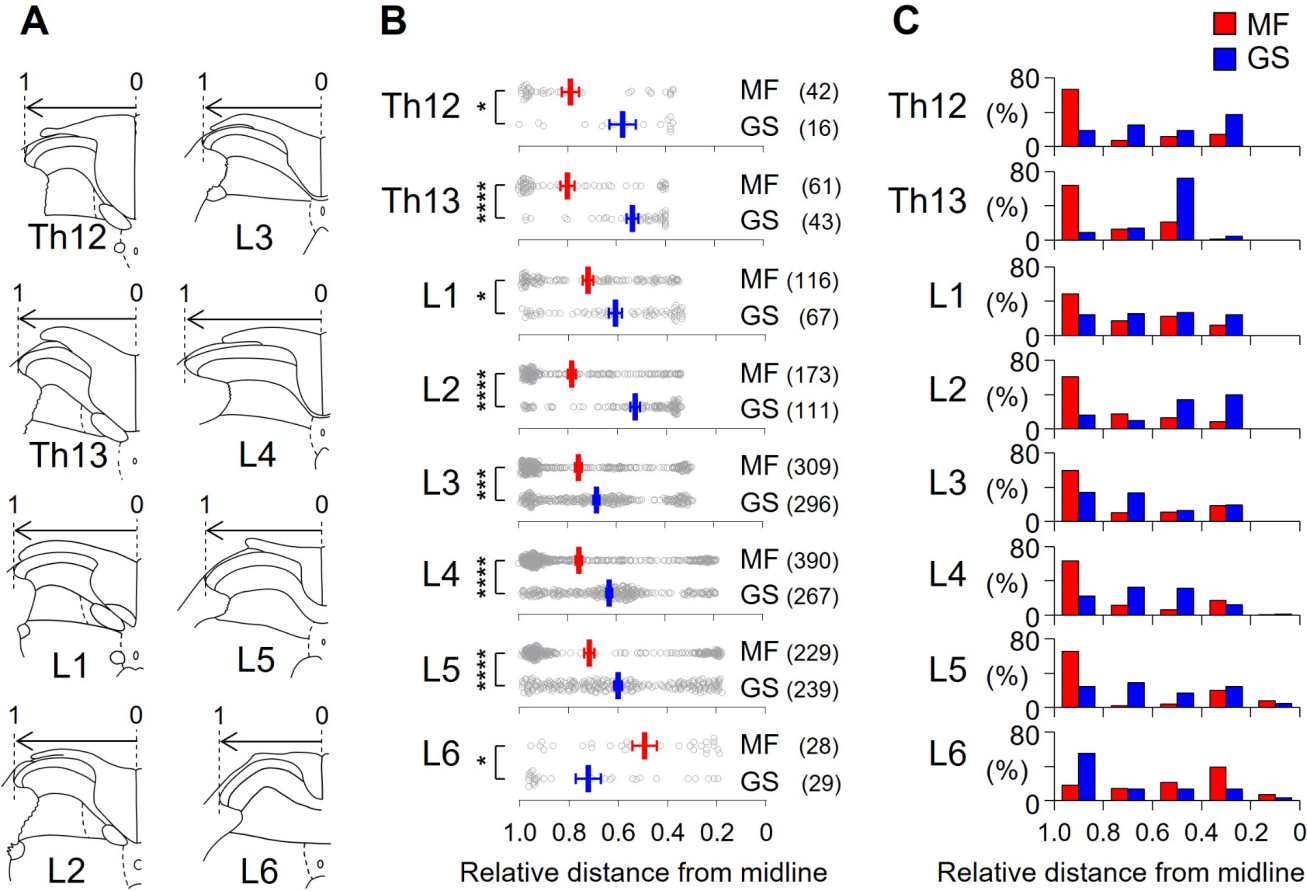
Mediolateral distribution of c-Fos-ir nuclei in the superficial DH after formalin injection into the MF or GS muscle. (**A**) Schemes used for data analysis according to the rat spinal cord atlas^28^: “0” = midline of the spinal cord (Th12–L6), “1” = most lateral border of the superficial DH (i.e., laminae I–II). (**B**) Relative distance of the c-Fos-ir nuclei from the midline. The data for analysis is derived from images provided in Fig. 2. MF: formalin injection into the MF; GS: formalin injection into the GS. The number of nuclei analyzed is shown in parenthesis. Note significantly different mediolateral distributions between the MF and GS (*p < 0.05, ***p < 0.001, and ****p < 0.0001, mixed-effects analysis (restricted maximum likelihood, REML) followed by Sidak’s multiple comparison test), showing that the MF input projected more laterally at the segments Th12–L5 compared to the GS input. Occasionally, the mediolateral input patterns were opposite at the segment L6, although the segment receives intense nociceptive input neither from the MF nor GS (see Fig. 2). (**C**) Proportion of c-Fos-ir nuclei in the mediolateral grid (spacing of 0.2) from midline of the spinal cord (“0”) to the most lateral border of the superficial DH (“1”).

**Fig. 4:**
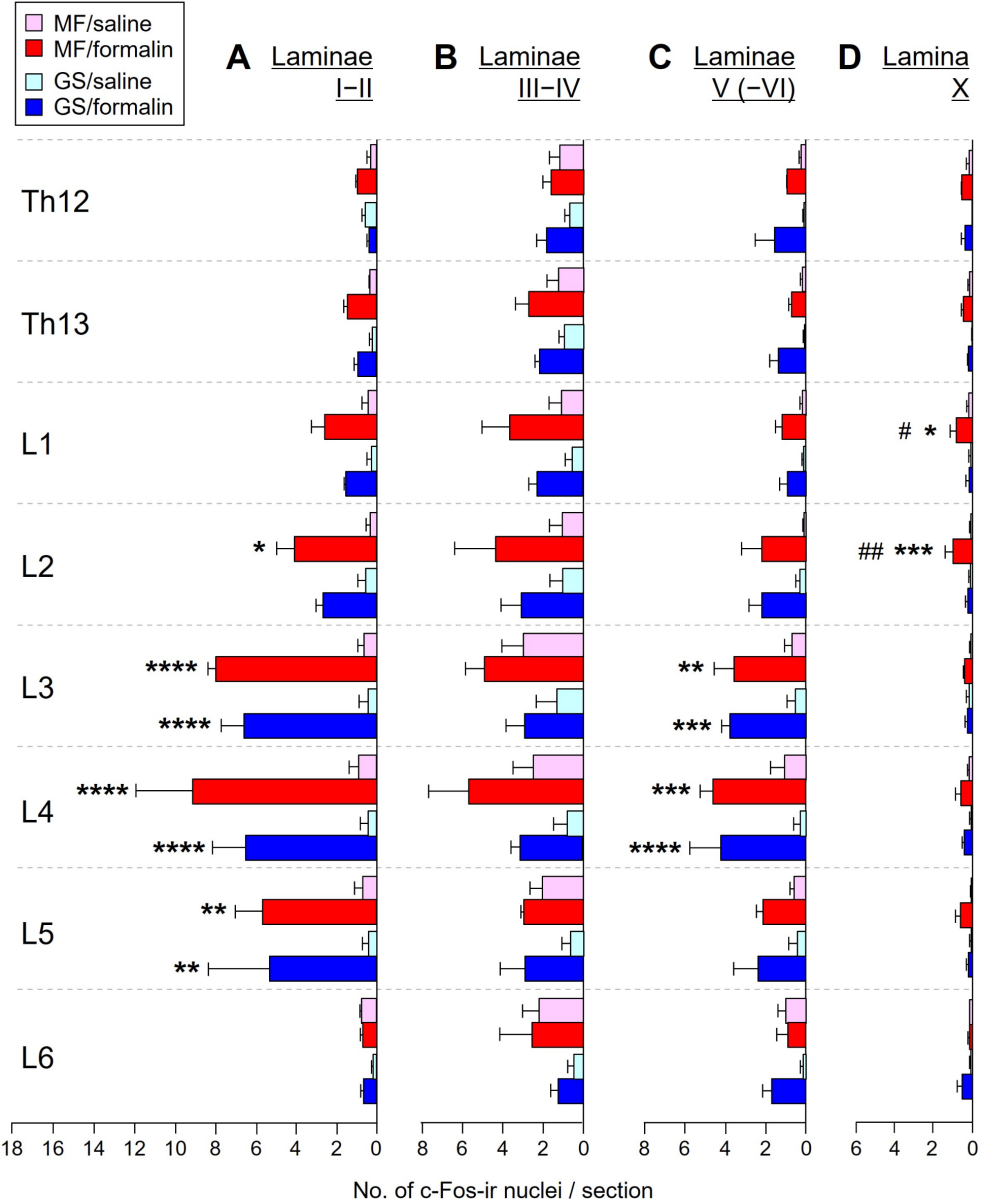
Number of c-Fos-ir nuclei in the DH after formalin or saline injection into the MF or GS muscle. (**A**) Number of c-Fos-ir nuclei per section in laminae I–II at the segments Th12–L6. (**B**) That in laminae III–IV. (**C**) That in laminae V (–VI). (**D**) That in lamina X (ipsilateral half from the midline). Data are derived from Fig. 2 and represented with mean + SEM of 3 rats. *p < 0.05, **p < 0.01, ***p < 0.001, and ****p < 0.0001, MF/formalin vs. MF/saline or GS/formalin vs. GS/saline, ^#^*p* < 0.05 and ^##^*p* < 0.01, MF/formalin vs. GS/formalin, two-way repeated measures ANOVA followed by Tukey’s multiple comparison test. See Supplementary Table 1 for statistical summary.

**Fig. 5:**
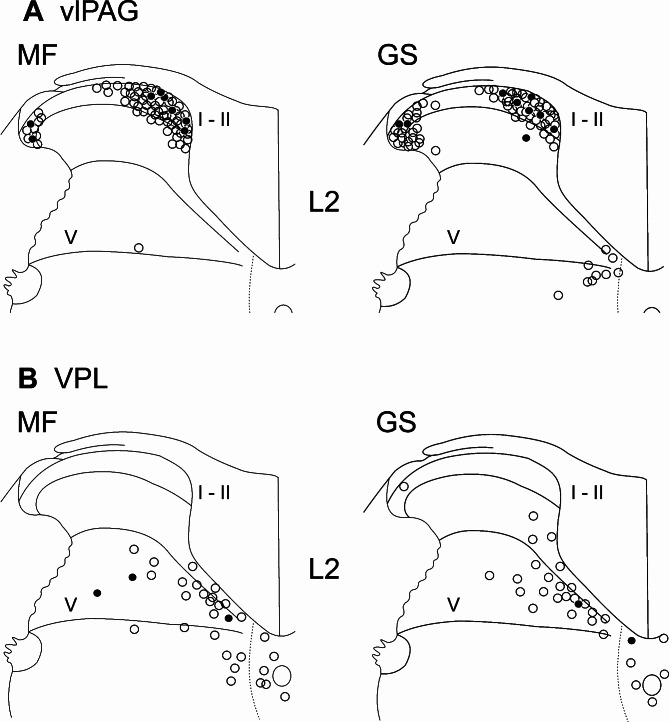
Distribution of double-labeled DH neurons. (**A**) Retrogradely labeled DH neurons at the lumbar segment L2 projecting to the vlPAG. (**B**) Those to the VPL. MF: formalin injection into the MF, GS: formalin injection into the GS. Open circles: DH neurons retrogradely labeled with FG but without c-Fos-ir nucleus (projection neurons without muscle input); filled circles: Those with FG and c-Fos-ir nucleus induced by the formalin injection (projection neurons with muscle input). In each panel, 75 sections from 5 rats were superimposed. Note that locations of double-labeled DH neurons as well as FG-positive ones projecting to the vlPAG and VPL were different: DH neurons projecting to the vlPAG were located in the superficial laminae, and those to the VPL were found only in the deep DH (laminae IV, V, (VI), VII, and X), while the numbers of neurons were small. The difference in the locations does not seem to be associated with input sources of the muscle (MF vs. GS).

**Fig. 6:**
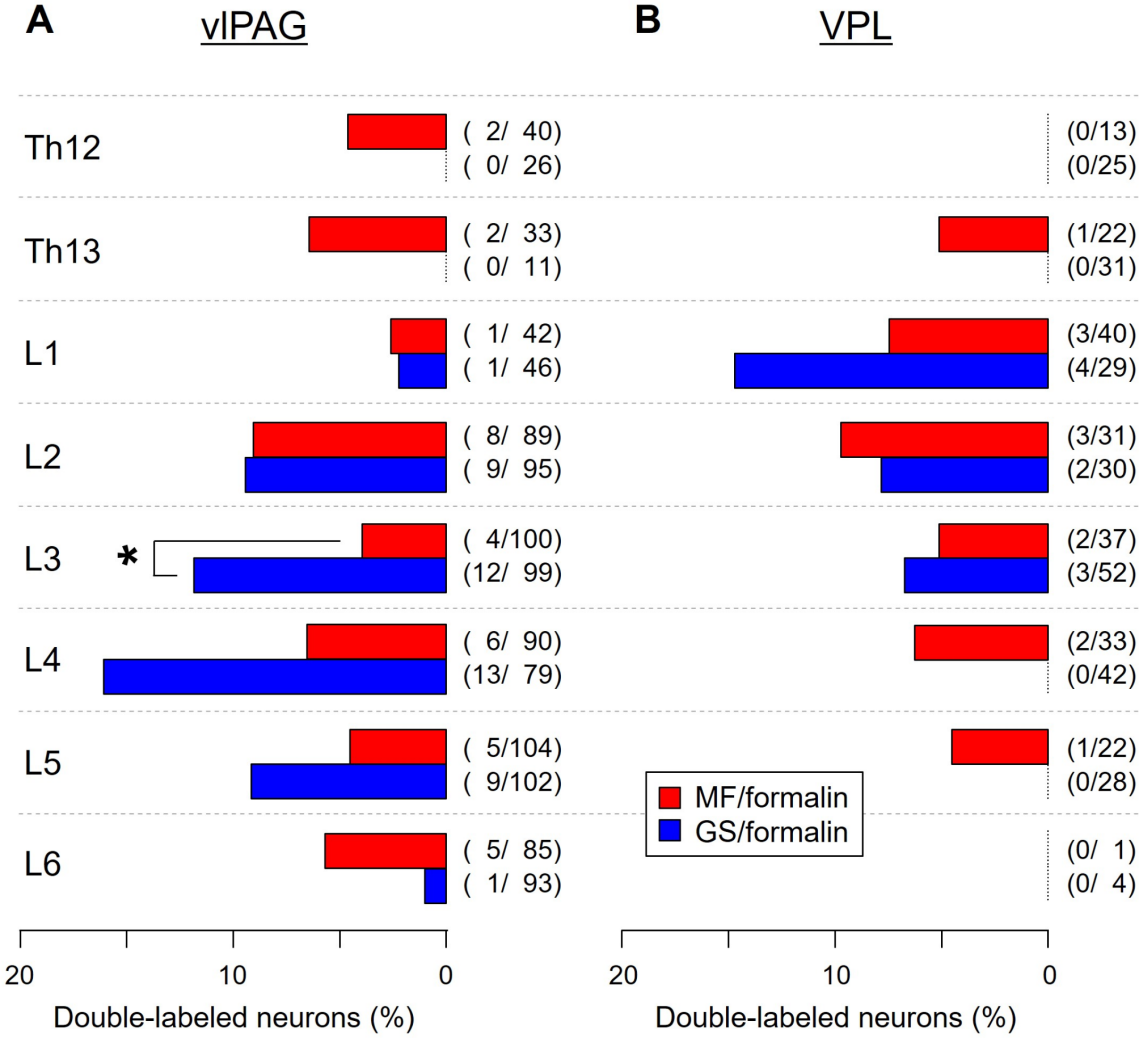
Segmental distribution of double-labeled DH neurons retrogradely stained with FG and showing c-Fos-ir nucleus. (**A**) Proportion of double-labeled neurons projecting to the vlPAG. (**B**) That to the VPL. The proportions were calculated by the number of double-labeled neurons out of the number of neurons with FG staining in the entire DH (i.e., laminae I–V (VI)) at the spinal segments Th12–L6. In all cases, 5% formalin was injected into the MF or GS muscle. *p < 0.05, Fisher’s exact probability test. Note the broader craniocaudal distribution of double-labeled DH neurons receiving nociceptive input from the MF compared to that from the GS, and the finding was true for both supraspinal centers (i.e., vlPAG and VPL).

In each of the Group 1 − 4 described above, three rats out of 5 were selected for quantitative analysis of c-Fos-ir nuclei (for Figs. 2, 3 and 4). For double-labeling experiments, five rats were used in each of the Group 1 − 8 (for Figs. 5 and 6). Ten to fifteen sections per rat from the segments Th12 − L6 were cut and analyzed. Every 5th section was processed to avoid double counts of nuclei. C-Fos-ir nuclei and FG in cytoplasm were visualized and counted using an excitation filter at wavelength of 578 nm and 350 nm, respectively, under an epifluorescence microscope (Zeiss Axioskope, Oberkochen, Germany) equipped with a digital CCD camera at a magnification of x400. Counting c-Fos-ir nuclei was made in the DH on the side ipsilateral to the formalin or saline injection. The relative location of c-Fos-ir nuclei in the grey matter was plotted on a schematic chart of the DH according to the description by Molander et al.^28^. Since the sections were not counterstained, the exact boundaries between the laminae were not discernible. Therefore, the DH was divided into three larger regions, namely, superficial DH (laminae I − II), nucleus proprius (laminae III–IV), neck of the DH (laminae V–VI in segments L3 − L6 or lamina V in segments Th12 − L2 as lamina VI is not present in these segments), and lamina X around the central canal (see Molander et al.^28^). The person responsible for counting the sections was blinded to the experimental condition.

### Statistical analysis

Data are represented as mean ± SEM. Relative locations of c-Fos-ir nuclei from the midline were analyzed using mixed-effects analysis followed by Sidak’s multiple comparison test. The number of c-Fos-ir nuclei was compared using two-way repeated measures analysis of variance (ANOVA) followed by Tukey’s multiple comparison test. Proportions of double-labeled neurons between the MF and GS were compared using Fisher’s exact probability test. A *p* < 0.05 (two-tailed) was regarded significant.

## Results

Figure 1 illustrates how DH neurons with muscle nociceptive input that projected to supraspinal centers were identified in this study. Fluorogold was stereotaxically injected as retrograde tracer into one of two supraspinal centers (vlPAG or VPL, Fig. 1A−D); injection site was verified histologically (Fig. 1E and F). In the DH, the retrograde tracer was identified in the cytoplasm (Fig. 1G). C-Fos immunoreactivity in response to formalin injection was used as a nuclear marker for DH neurons with muscle nociceptor input (Fig. 1H). Double-labeled neurons were interpreted to have both muscle nociceptive input and projection to vlPAG or VPL. Figure 1E − H are examples for Fig. 1A (i.e., FG injection into vlPAG and c-Fos-ir induced by nociceptive input from low back multifidus).

### Distribution of c-Fos-Ir nuclei in the DH

Using c-Fos immunohistochemistry, we examined whether somatotopic representation of nociceptive input to the spinal DH could differ between the MF and GS, with particular regard to the signal intensity and projection pattern. Formalin injection into the muscles induced discernible c-Fos-ir nuclei especially in the superficial DH (laminae I − II), that was adjacent to the posterior funiculi. When we superimposed the distribution of c-Fos-ir nuclei obtained from all sections analyzed (36–45 sections from 3 rats), somatotopic representation of nociceptive input to the DH could be better visualized (Fig. 2): (1) Compared to the saline injected group, formalin injection into the MF and GS induced much more c-Fos-ir nuclei across all DH laminae (i.e., laminae I − V (VI)) at all spinal segments analyzed (Th12 − L6). (2) Formalin injection into the MF induced discernible c-Fos expression especially in the most lateral area of the superficial DH (laminae I − II) with the wider craniocaudal extension of Th12 − L5 (indicated by arrows, Fig. 2A). (3) Formalin injection into the GS induced discernible c-Fos expression especially in the middle area of the superficial DH (laminae I − II) with the craniocaudal distribution of L3 − L5 (indicated by arrows, Fig. 2B). (4) c-Fos expression appeared to be dense around the central canal corresponding to lamina X in the MF/formalin and GS/formalin groups compared to the MF/saline and GS/saline groups, respectively (Fig. 2A and B). Lamina VII was not covered by our digital images.

Since mediolateral and craniocaudal (segmental) distributions of c-Fos-ir nuclei in laminae I − II were different between the MF and GS as described above, we calculated the relative distance of the location of c-Fos-ir nuclei from the midline of the spinal cord (Th12 − L6, Fig. 3A). In the calculation, the lateral tip of the superficial DH was defined as “1”, and the relative location of a c-Fos-ir nucleus was indicated as a fraction of 1 with the origin of “0” placed at the midline of the spinal cord.

The relative distance appeared to be different between the MF and GS (Fig. 3B). Mixed-effects analysis (restricted maximum likelihood, REML) revealed significant effects for SEGMENT (F_5.626, 1379_ = 4.263, *p* = 0.0004), MUSCLE (F_1, 684_ = 51.62, *p* < 0.0001), and SEGMENT × MUSCLE interaction (F_7, 1716_ = 9.477, *p* < 0.0001). The *post-hoc* test (Sidak’s multiple comparison test) showed significant differences between the MF and GS (*p* = 0.0195, *p* < 0.0001, *p* = 0.0109, *p* < 0.0001, *p* = 0.0008, *p* < 0.0001, *p* < 0.0001, and *p* = 0.0174 at the spinal segment Th12, Th13, L1, L2, L3, L4, L5, and L6, respectively). The statistical results demonstrated that the MF input to the superficial DH projected more laterally at the segments Th12–L5 compared to the GS input. As an exception to this pattern, the mediolateral input distributions were opposite at the segment L6, but the segment receives intense nociceptive input neither from the MF nor GS (also see Fig. 2).

To compare the mediolateral difference or overlap of DH neurons activated by muscle nociceptive stimuli, proportion of c-Fos-ir nuclei was calculated using a grid from midline of the spinal cord (“0”) to the most lateral border of the superficial DH (“1”) in intervals of 0.2 (Fig. 3C). The proportion of DH neurons activated by nociceptive MF stimuli was higher in the most lateral grid (0.8 ~ 1.0) and that activated by nociceptive GS stimuli in the middle grid (0.2 ~ 0.8), while the mediolateral distribution in the grid had some overlaps.

To examine the signal intensity of nociceptive input from the MF and GS, the number of c-Fos-ir nuclei in the DH laminae I − II, III − IV, V (–VI), and X was counted and analyzed. Overall, the number of c-Fos-positive nuclei was higher in the formalin injected groups (MF/formalin and GS/formalin) than in the saline injected groups (MF/saline and GS/saline), across all DH laminae at segments Th12–L5 (Fig. 4). The highest number of c-Fos-ir nuclei was found in laminae I − II (Fig. 4A) with a progressive decline in laminae III − IV (Fig. 4B) and V (–VI) (Fig. 4C). In laminae I − II, significant differences between the MF/formalin and MF/saline groups were detected at segments L2–L5, while those between the GS/formalin and GS/saline groups were detected at segments L3–L5 (*p* < 0.05 ~ 0.0001, two-way repeated measures ANOVA followed by Tukey’s multiple comparison test, Fig. 4A and supplementary Table 1). No significant difference among the four groups was detected in laminae III − IV at any segments analyzed (Fig. 4B and supplementary Table 1). In laminae V (− VI), significant differences between the MF/formalin and MF/saline groups and between the GS/formalin and GS/saline groups were detected at segments L3 and L4 (*p* < 0.01 ~ 0.0001, Fig. 4C and supplementary Table 1). When comparing the data between the MF/formalin and GS/formalin groups and between the MF/saline and GS/saline groups, no significant difference was detected at any DH laminae and segments analyzed (Fig. 4A − C and supplementary Table 1).

In lamina X, the number of c-Fos-ir nuclei for the MF/formalin groups was significantly higher than that of MF/saline groups and GS/formalin groups at segments L1 and L2 (*p* < 0.05 ~ 0.001, Fig. 4D and supplementary Table 1).

### Supraspinal projections of DH neurons to the vlPAG and VPL

To examine whether supraspinal projections of nociceptive input could differ between the low back and limb muscles, we analyzed the distribution of double-labeled DH neurons activated by nociceptive stimuli to the MF or GS (i.e., c-Fos-ir) and projecting to the vlPAG or VPL (i.e., FG-positive). The distribution at the segment L2 was exemplified in Fig. 5. Interestingly, the locations of DH neurons projecting to the vlPAG or VPL were different: Most neurons projecting to the vlPAG were located in the superficial laminae of the DH, with just a few in the deep laminae (Fig. 5A), whereas neurons projecting to the VPL were found only in the deep DH (laminae IV, V, (VI), VII, and X; Fig. 5B). The double-labeled DH neurons were restricted to the side ipsilateral to the formalin injection (i.e., on the side contralateral to the FG injection). The different locations do not seem to be associated with the input source of the muscles (MF or GS).

With regard to the segmental distribution, DH neurons projecting to the vlPAG and VPL in the MF/formalin and GS/formalin groups widely distributed across the spinal segments Th12–L6 analyzed (Fig. 6). However, DH neurons projecting to the vlPAG with input from the MF widely existed in all spinal segments analyzed (Th12–L6), whereas those with input from the GS were present only in segments L1 − L6, peaking at L4 (Fig. 6A). At the segment L3, proportion of the DH neurons was significantly higher in the GS/formalin group than in the MF/formalin group (*p* < 0.05, Fisher’s exact probability test, Fig. 6A). Likewise, DH neurons projecting to the VPL with input from the MF widely existed in segments Th13–L5, whereas those with input from the GS were present only in segments L1 − L3 (Fig. 6B). Thus, supraspinally projecting neurons receiving nociceptive input from the MF had the wider craniocaudal extension compared with those from the GS.

## Discussion

Using c-Fos immunohistochemistry combined with retrograde FG tracing of DH neurons in this systematic study, we found that spinal projection patterns of nociceptive input from the MF (lateral) were considerably different from those of GS (middle/medial) revealing highly organized somatotopic arrangements of deep tissue representation in the spinal DH. With regard to the craniocaudal (segmental) distributions of the projection neurons, supraspinal projection originated from more segments for MF than GS muscles were somehow different, although the overall number of double-labeled DH neurons projecting to the vlPAG and VPL was small.

### Spinal projections of nociceptive input from MF and GS

It has been well documented that primary afferent terminals originating in the skin exhibited highly organized somatotopic representation in the spinal DH in previous studies^29,30^. In the present systematic study, DH neurons activated by nociceptive stimuli to the MF and GS displayed a distinct mediolateral and craniocaudal (segmental) somatotopy: DH neurons activated by MF stimuli were located in the most lateral area of laminae I − II at the spinal segments Th12 − L5, and those with GS input were located in the medial area of laminae I − II at segments L3 − L5. The number of c-Fos-ir nuclei did not differ between the two muscles in any DH laminae (I − II, III − IV, and V (− VI)) and segments (Th12 − L6) analyzed in this experiment.

### Mediolateral distribution of the spinal projections

The lateral preference of nociceptive input from the MF was consistent with a previous study on sensory fibers labeled with horseradish peroxidase (HRP): fibers in the dorsal ramus of a rat thoracic nerve projected to the lateral third of the superficial DH, while those in the ventral ramus projected more medially^31^. A direct comparison of DH representation of axial vs. limb muscles has not been reported, but data for individual muscles are consistent with the pattern reported here. MF muscle representation in our preliminary study^16^ (only lumbar segments L3 and L5), as well as dorsal neck muscle representation (in response to injections of nerve growth factor in mice^32^ and capsaicin in cats^33^) have been shown to be in the lateral part of the superficial DH. On the other hand, axons innervating the GS projected to the middle to medial area of the superficial DH of guinea pigs^34^ and rats^35,36^. Inflammation induced by intramuscular injection of turpentine oil into the rat GS also resulted in c-Fos expression in the middle of the superficial DH^37^. Thus, mediolateral representation of nociceptive input from the MF and GS was distinctly organized in the superficial DH. Muscle nociceptive input followed the same pattern as cutaneous nociceptive input with proximal body parts being represented in lateral DH^31^. This distinct somatotopy might permit discrimination of pain arising from the two muscles, provided that at least some of these DH neurons project to cortex via VPL.

### Segmental (craniocaudal) distribution of the spinal projections

The nociceptive input from the MF was distributed widely in the craniocaudal direction, which is consistent with its more extensive representation in the ventral horn as part of postural control. In a previous study, a neuronal tracer wheat germ agglutinin (WGA)-HRP conjugate, which preferentially labels small-diameter afferents, was injected into the MF between spinous processes L4 − L5 and L5 − L6 of two cats^38^. The WGA-HRP reaction product was observed as far cranial as the segment Th10 in one cat and C8 in the other cat. On the other hand, reaction product of an HRP cocktail applied to nerves innervating the GS was observed at segments L3 − L5 ^35^. C-fiber afferents in the GS of guinea pigs have been reported to project with the craniocaudal extension of two to three segments^34^. The craniocaudal extension of a few segments was compatible with the segmental distribution of c-Fos-ir nuclei observed in this study for the MF muscle.

While cutaneous pain is sharp and localized and visceral pain is blunt and poorly localized, muscle pain has intermediate sensory characteristics between the skin and visceral pains^3^. The difference in segmental integration of nociceptive input between MF and GS muscles may explain why low back muscle pain is often widespread while pain in limb muscles is mostly localized.

### Supraspinal nociceptive processing via projecting DH neurons activated by MF and GS stimuli

To date, several brain regions have been discussed as critical targets or pathways responsible for the perception of muscle pain^15,18–21^. However, spinal signal processing of muscle nociceptor input has mostly been reported for interneurons in the DH, but not for projection neurons^39^. Since supraspinal processing of muscle pain is only possible via such elusive projection neurons, we performed a systematic search for projecting DH neurons activated by muscle nociceptive stimuli, using double-labeling of DH neurons (c-Fos for muscle nociceptive input, fluorogold for projection to two important supraspinal centers). We observed the following findings in the present study: (1) The DH neurons projecting to the vlPAG were located in the superficial, but not deep, DH, while those projecting to the VPL were in the deep, but not superficial, DH. (2) The same patterns were true for double-labeled DH neurons activated by muscle nociceptive stimuli. (3) Craniocaudal (segmental) distributions of DH neurons projecting to the vlPAG or VPL were more extensive for input from the MF compared to that from the GS, although the overall number of DH neurons projecting to the two supraspinal centers was small.

The superficial location of double-labeled neurons in the DH for the vlPAG and deep location for the VPL fit well with previous studies^40–42^. The wide craniocaudal spread of the DH projection neurons with input from the MF likely reflects the broader craniocaudal distribution of c-Fos-ir nuclei in the spinal DH elicited by nociceptive input from the MF compared to that from the GS (see section “*Spinal projections of nociceptive input from MF and GS*”). Projection neurons in rats are present in lamina I and scattered throughout laminae III–VI, with very few found in lamina II at lumbar segments^39^. The proportion of projection neurons in the total neuronal population in lamina I at the L4 segment is less than 5%, and the lamina I projection neurons have connections mainly to the caudal ventrolateral medulla (87.5%), solitary tract nucleus (22.5%), lateral parabrachial area (95%), PAG (30%), and certain nuclei in the thalamus (3.8%)^39^. Thus, the proportion of DH neurons activated by muscle nociceptive stimuli and projecting to the vlPAG and VPL is predicted to be low. The small number of DH projection neurons with muscle nociceptive inputs fits well with a previous study that showed few DH neurons with input from cutaneous, articular, and visceral afferents projecting to several supraspinal sites^43^.

The number of DH neurons activated by muscle nociceptive stimuli and projecting to the vlPAG and VPL was found to be small, which may explain why previous studies overlooked these small populations. In summary, we were able to demonstrate that nociceptive signals originating in low back or limb muscles are not only processed in DH interneurons, but also forwarded to two important supraspinal regions.

### Ventrolateral PAG as a supraspinal center processing muscle nociception

The mesencephalic PAG is one of the supraspinal centers responsible for the processing of muscle pain, by integrating ascending sensory information with descending inhibition and facilitation derived from the medial nociceptive system (prefrontal cortex, anterior cingulate cortex, and insula) involved in affective-motivational aspects of pain^44–46^. In the rat PAG, noxious stimulation to the deep neck muscles induced c-Fos expression predominantly in the ventrolateral area (i.e., vlPAG), while cutaneous noxious stimulation in the lateral PAG^19,47^. Intramuscular injection of 5% formalin into the rat MF induced higher c-Fos expression in the piriform cortex, nucleus accumbens, basolateral nucleus of amygdala, paraventricular hypothalamic nucleus, ventral tegmental area, and vlPAG^15^. The vlPAG is known as a crucial brain region that activates descending pain inhibitory system and modulates nociception/pain and anxiety ^44–46,48−50^ and descending pain inhibition has been reported to be driven strongly with input to DH neurons from muscle nociceptors compared to that from cutaneous ones^3^. Optogenetic activation of excitatory descending neural pathway from the dorsomedial prefrontal cortex to the vlPAG has been shown to produce analgesic and anxiolytic effects in a mouse model of chronic pain^51^. In addition, the vlPAG is assumed to cause decreased autonomic functions, such as bradycardia and hypotension, which are observed in patients with chronic muscle pain^3^ and in rats subjected to experimental muscle pain^20,52^. Approximately 25% of c-Fos-ir neurons in the vlPAG, which were evoked by intramuscular injection of formalin into the deep dorsal neck muscles, have been reported to project to the rostral ventrolateral medulla (RVLM), suggesting that the direct vlPAG-RVLM pathway could mediate the deep pain-evoked autonomic hypofunction^20^. In summary, both low back and limb muscles provide input to nociceptive signal integration in the vlPAG, but MF input is integrated across a broader cranio-caudal extent. Thus, nociceptive input from axial muscles is processed in a way consistent with its motor function in postural control via the medial motor system.

### VPL as a supraspinal center processing muscle nociception

Neurons in the VPL and spinothalamic tract have long been discussed as a supraspinal component responsible for the perception of muscle pain. In a recent study, a subclass of spinothalamic cells within the anterolateral pathway, that are located in the medial part of the deep DH and project to the rostral part of the VPL, has been identified in mice^53^. Activation of the cells did not influence nociceptive behaviors, but inhibition of the cells caused deficits in coordination of movement, suggesting that these spinothalamic cells in the ventral pathway have little to do with pain^53^.

In an earlier experiment, cells in the spinothalamic tract have been shown to be activated by chemical stimulation of fine afferents in the triceps surae muscles of anesthetized monkeys^54^. Kniffki and Mizumura have reported the existence of neurons in the VPL and in the transition zone between the VPL and ventrolateral nucleus (VL) to be activated by nociceptive input from the cat GS^21^. In rats, activation of tetrodotoxin-resistant nociceptive afferents in the GS nerve increased c-Fos expression in the VL and VPL^18^. The neuronal activation in the VPL of the lateral system may mediate the sensory-discriminative component of muscle pain^55^. Different from the GS in the hindlimb, no prominent increase in the number of c-Fos-ir nuclei had been observed in the VPL and the ventral posteromedial nucleus of the thalamus (VPM) after intramuscular formalin injection into the MF in the low back^15^. These observations have been interpreted to suggest that nociceptive information originating from the MF is processed in other thalamic nuclei of the medial system, consistent with the strong emotional-affective component of low back pain and its affection by psychosocial factors^7–9^. However, our data unequivocally show (via double-labeling) that some deep DH projection neurons forward nociceptive information from MF to the lateral pain system also.

### Technical considerations

The present study provided a fraction of the whole picture. We should be careful when interpreting the data in this study: (1) Not all nociceptors in the MF and GS were activated by the formalin injection. (2) Diffusion conditions of formalin solution, innervation densities of nociceptors, synaptic efficacies of nociceptive connections in the DH, and other factors might be different. (3) FG was probably not picked up by all neurons in the vlPAG and VPL. (4) FG may have been picked up by axons passing through the injection sites.

### Limitations

In the present study, we used only male rats. However, epidemiologic, clinical, and experimental evidence report female predominance in musculoskeletal pain^56,57^. Higher pressure- and glutamate-evoked pain sensitivity have been shown in muscles of female than male subjects^58,59^. In rats, muscular mechanical withdrawal threshold was lower in female^60^ and glutamate-evoked masseter muscle afferent activity was greater in female than in male^59^. These studies indicate that sexual dimorphism influences the levels of activity in central projections of muscle nociceptive input.

## Conclusions

Using neuroanatomical tracing experiments, we found that nociceptive pathways originating from the MF and GS to the spinal DH were considerably different with highly organized somatotopic distributions. The lateral location of superficial DH neurons receiving low back muscle input is consistent with the medio-lateral somatotopy of cutaneous DH inputs, while it is opposite to the somatotopy in the ventral horn, where axial muscles are controlled via the medial motor system. Craniocaudal distributions of DH neurons receiving low back muscle input and projecting to the vlPAG and VPL appeared to be wider than for limb muscle input. This sensory representation is consistent with the higher segmental integration of the medial motor system. Via vlPAG low back muscle pain may also have more widespread effects on autonomic nervous system functions. Via VPL, the higher segmental integration of low back muscle input may explain why low back pain is often widespread and less well localized than limb muscle pain.

## Electronic supplementary material

Below is the link to the electronic supplementary material.

Supplementary Material 1.

## Data Availability

Data is provided within the manuscript or supplementary information files.

## References

[1] Hoy, D. et al. The global burden of low back pain: Estimates from the global burden of Disease 2010 study. *Ann. Rheum. Dis.* **73**, 968–974. 10.1136/annrheumdis-2013-204428 (2014). 24665116 10.1136/annrheumdis-2013-204428

[2] Perrot, S. et al. The IASP classification of chronic pain for ICD-11: Chronic secondary musculoskeletal pain. *Pain* **160**, 77–82. 10.1097/j.pain.0000000000001389 (2019). 30586074 10.1097/j.pain.0000000000001389

[3] Mense, S. Nociception from skeletal muscle in relation to clinical muscle pain. *Pain* **54**, 241–289. 10.1016/0304-3959(93)90027-M (1993). 8233542 10.1016/0304-3959(93)90027-M

[4] Nakamura, M., Nishiwaki, Y., Ushida, T. & Toyama, Y. Prevalence and characteristics of chronic musculoskeletal pain in Japan. *J. Orthop. Sci.* **16**, 424–432. 10.1007/s00776-011-0102-y (2011). 21678085 10.1007/s00776-011-0102-y PMC3140943

[5] Kellgren, J. H. The anatomical source of back pain. *Rheumatol. Rehabil*. **16**, 3–12. 10.1093/rheumatology/16.1.3 (1977). 139667 10.1093/rheumatology/16.1.3

[6] Knezevic, N. N., Candido, K. D., Vlaeyen, J. W. S., Van Zundert, J. & Cohen, S. P. Low back pain. *Lancet* **398**, 78–92. 10.1016/S0140-6736(21)00733-9 (2021). 34115979 10.1016/S0140-6736(21)00733-9

[7] Fernandez, E. & Turk, D. C. Sensory and affective components of pain: Separation and synthesis. *Psychol. Bull.* **112**, 205–217. 10.1037/0033-2909.112.2.205 (1992). 1454892 10.1037/0033-2909.112.2.205

[8] Koleck, M., Mazaux, J. M., Rascle, N. & Bruchon-Schweitzer, M. Psycho-social factors and coping strategies as predictors of chronic evolution and quality of life in patients with low back pain: A prospective study. *Eur. J. Pain* **10**, 1–11. 10.1016/j.ejpain.2005.01.003 (2006). 16291293 10.1016/j.ejpain.2005.01.003

[9] Matsudaira, K. et al. Assessment of psychosocial risk factors for the development of non-specific chronic disabling low back pain in Japanese workers-findings from the Japan Epidemiological Research of Occupation-related Back Pain (JOB) study. *Ind. Health*. **53**, 368–377. 10.2486/indhealth.2014-0260 (2015). 26051289 10.2486/indhealth.2014-0260 PMC4551067

[10] Mannion, A. F. et al. Muscle fibre size and type distribution in thoracic and lumbar regions of erector spinae in healthy subjects without low back pain: Normal values and sex differences. *J. Anat.* **190** (Pt 4), 505–513. 10.1046/j.1469-7580.1997.19040505.x (1997). 9183674 10.1046/j.1469-7580.1997.19040505.x PMC1467636

[11] Staron, R. S. et al. Fiber type composition of the vastus lateralis muscle of young men and women. *J. Histochem. Cytochem.* **48**, 623–629. 10.1177/002215540004800506 (2000). 10769046 10.1177/002215540004800506

[12] Kuypers, H. G. J. M. Anatomy of the descending pathways. (ed. Brooks, V.B.) Handbook of physiology, Sect. 1: The nervous system, Vol. 2: Motor control, Part I. 597–666 (American Physiological Society, Bethesda, 1981).

[13] Lemon, R. N. Descending pathways in motor control. *Annu. Rev. Neurosci.* **31**, 195–218. 10.1146/annurev.neuro.31.060407.125547 (2008). 18558853 10.1146/annurev.neuro.31.060407.125547

[14] Hoheisel, U., Taguchi, T., Treede, R. D. & Mense, S. Nociceptive input from the rat thoracolumbar fascia to lumbar dorsal horn neurones. *Eur. J. Pain* **15**, 810–815. 10.1016/j.ejpain.2011.01.007 (2011). 21330175 10.1016/j.ejpain.2011.01.007

[15] Ohtori, S. et al. Fos expression in the rat brain and spinal cord evoked by noxious stimulation to low back muscle and skin. *Spine* **25**, 2425–2430. 10.1097/00007632-200010010-00002 (2000). 11013492 10.1097/00007632-200010010-00002

[16] Taguchi, T., John, V., Hoheisel, U. & Mense, S. Neuroanatomical pathway of nociception originating in a low back muscle (multifidus) in the rat. *Neurosci. Lett.* **427**, 22–27. 10.1016/j.neulet.2007.08.021 (2007). 17928140 10.1016/j.neulet.2007.08.021

[17] Taguchi, T., Hoheisel, U. & Mense, S. Dorsal horn neurons having input from low back structures in rats. *Pain* **138**, 119–129. 10.1016/j.pain.2007.11.015 (2008). 18164133 10.1016/j.pain.2007.11.015

[18] Gholami, S., Lambertz, D., Hoheisel, U. & Mense, S. Effects on c-Fos expression in the PAG and thalamus by selective input via tetrodotoxin-resistant afferent fibres from muscle and skin. *Neurosci. Res.* **56**, 270–278. 10.1016/j.neures.2006.07.004 (2006). 16962193 10.1016/j.neures.2006.07.004

[19] Keay, K. A. & Bandler, R. Deep and superficial noxious stimulation increases Fos-like immunoreactivity in different regions of the midbrain periaqueductal grey of the rat. *Neurosci. Lett.* **154**, 23–26. 10.1016/0304-3940(93)90162-e (1993). 8361643 10.1016/0304-3940(93)90162-e

[20] Keay, K. A., Li, Q. F. & Bandler, R. Muscle pain activates a direct projection from ventrolateral periaqueductal gray to rostral ventrolateral medulla in rats. *Neurosci. Lett.* **290**, 157–160. 10.1016/s0304-3940(00)01329-x (2000). 10963887 10.1016/s0304-3940(00)01329-x

[21] Kniffki, K. D. & Mizumura, K. Responses of neurons in VPL and VPL-VL region of the cat to algesic stimulation of muscle and tendon. *J. Neurophysiol.* **49**, 649–661. 10.1152/jn.1983.49.3.649 (1983). 6834092 10.1152/jn.1983.49.3.649

[22] Zimmermann, M. Ethical guidelines for investigations of experimental pain in conscious animals. *Pain* **16**, 109–110. 10.1016/0304-3959(83)90201-4 (1983). 6877845 10.1016/0304-3959(83)90201-4

[23] Percie du Sert. The ARRIVE guidelines 2.0: Updated guidelines for reporting animal research. *PLoS Biol.* **18**, e3000410. 10.1371/journal.pbio.3000410 (2020). 32663219 10.1371/journal.pbio.3000410 PMC7360023

[24] Paxinos, G. & Watson, C. *The Rat Brain in Stereotaxic Coordinates* (Academic, 1986). 10.1016/0165-0270(80)90021-7 6110810

[25] Coggeshall, R. E. Fos, nociception and the dorsal horn. *Prog. Neurobiol.* **77**, 299–352. 10.1016/j.pneurobio.2005.11.002 (2005). 16356622 10.1016/j.pneurobio.2005.11.002

[26] Sauvage, M., Kitsukawa, T. & Atucha, E. Single-cell memory trace imaging with immediate-early genes. *J. Neurosci. Methods*. **326**, 108368. 10.1016/j.jneumeth.2019.108368 (2019). 31356836 10.1016/j.jneumeth.2019.108368

[27] West, A. E., Griffith, E. C. & Greenberg, M. E. Regulation of transcription factors by neuronal activity. *Nat. Rev. Neurosci.* **3**, 921–931. 10.1038/nrn987 (2002). 12461549 10.1038/nrn987

[28] Molander, C., Xu, Q. & Grant, G. The cytoarchitectonic organization of the spinal cord in the rat. I. The lower thoracic and lumbosacral cord. *J. Comp. Neurol.* **230**, 133–141. 10.1002/cne.902300112 (1984). 6512014 10.1002/cne.902300112

[29] Swett, J. E. & Woolf, C. J. The somatotopic organization of primary afferent terminals in the superficial laminae of the dorsal horn of the rat spinal cord. *J. Comp. Neurol.* **231**, 66–77. 10.1002/cne.902310106 (1985). 3968229 10.1002/cne.902310106

[30] Woolf, C. J. & Fitzgerald, M. Somatotopic organization of cutaneous afferent terminals and dorsal horn neuronal receptive fields in the superficial and deep laminae of the rat lumbar spinal cord. *J. Comp. Neurol.* **251**, 517–531. 10.1002/cne.902510407 (1986). 3782502 10.1002/cne.902510407

[31] Grant, G. Projection patterns of primary sensory neurons studied by transganglionic methods: somatotopy and target-related organization. *Brain Res. Bull.* **30**, 199–208. 10.1016/0361-9230(93)90245-7 (1993). 8457868 10.1016/0361-9230(93)90245-7

[32] Panfil, C., Makowska, A. & Ellrich, J. Brainstem and cervical spinal cord Fos immunoreactivity evoked by nerve growth factor injection into neck muscles in mice. *Cephalalgia* **26**, 128–135. 10.1111/j.1468-2982.2005.01005.x (2006). 16426266 10.1111/j.1468-2982.2005.01005.x

[33] Pilyavskii, A. I. et al. Capsaicin-induced effects on c-fos expression and NADPH-diaphorase activity in the feline spinal cord. *Eur. J. Pharmacol.* **521**, 70–78. 10.1016/j.ejphar.2005.08.006 (2005). 16168409 10.1016/j.ejphar.2005.08.006

[34] Ling, L. J. et al. Central projection of unmyelinated (C) primary afferent fibers from gastrocnemius muscle in the guinea pig. *J. Comp. Neurol.* **461**, 140–150. 10.1002/cne.10619 (2003). 12724833 10.1002/cne.10619

[35] Panneton, W. M., Gan, Q. & Juric, R. The central termination of sensory fibers from nerves to the gastrocnemius muscle of the rat. *Neuroscience* **134**, 175–187. 10.1016/j.neuroscience.2005.02.032 (2005). 15953682 10.1016/j.neuroscience.2005.02.032

[36] Panneton, W. M., Gan, Q. & Ariel, M. Injections of algesic solutions into muscle activate the lateral reticular formation: A nociceptive relay of the spinoreticulothalamic tract. *PLoS One* **10**, e0130939. 10.1371/journal.pone.0130939 (2015). 26154308 10.1371/journal.pone.0130939 PMC4496070

[37] Hu, J. Y. & Zhao, Z. Q. Differential contributions of NMDA and non-NMDA receptors to spinal Fos expression evoked by superficial tissue and muscle inflammation in the rat. *Neuroscience* **106**, 823–831. 10.1016/s0306-4522(01)00299-8 (2001). 11682167 10.1016/s0306-4522(01)00299-8

[38] Gillette, R. G., Kramis, R. C. & Roberts, W. J. Spinal projections of cat primary afferent fibers innervating lumbar facet joints and multifidus muscle. *Neurosci. Lett.* **157**, 67–71. 10.1016/0304-3940(93)90644-z (1993). 7694191 10.1016/0304-3940(93)90644-z

[39] Todd, A. J. Neuronal circuitry for pain processing in the dorsal horn. *Nat. Rev. Neurosci.* **11**, 823–836. 10.1038/nrn2947 (2010). 21068766 10.1038/nrn2947 PMC3277941

[40] Wiberg, M. & Blomqvist, A. The spinomesencephalic tract in the cat: Its cells of origin and termination pattern as demonstrated by the intraaxonal transport method. *Brain Res.* **291**, 1–18. 10.1016/0006-8993(84)90645-0 (1984). 6697174 10.1016/0006-8993(84)90645-0

[41] Menetrey, D. & De Pommery, J. Origins of spinal ascending pathways that reach central areas involved in visceroception and visceronociception in the rat. *Eur. J. Neurosci.* **3**, 249–259. 10.1111/j.1460-9568.1991.tb00087.x (1991). 12106203 10.1111/j.1460-9568.1991.tb00087.x

[42] Kim, H., Cui, L., Kim, J. & Kim, S. J. Transient receptor potential vanilloid type 1 receptor regulates glutamatergic synaptic inputs to the spinothalamic tract neurons of the spinal cord deep dorsal horn. *Neuroscience* **160**, 508–516. 10.1016/j.neuroscience.2009.02.019 (2009). 19236908 10.1016/j.neuroscience.2009.02.019

[43] Menetrey, D., Gannon, A., Levine, J. D. & Basbaum, A. I. Expression of c-fos protein in interneurons and projection neurons of the rat spinal cord in response to noxious somatic, articular, and visceral stimulation. *J. Comp. Neurol.* **285**, 177–195. 10.1002/cne.902850203 (1989). 2503547 10.1002/cne.902850203

[44] Lei, J., Sun, T., Lumb, B. M. & You, H. J. Roles of the periaqueductal gray in descending facilitatory and inhibitory controls of intramuscular hypertonic saline induced muscle nociception. *Exp. Neurol.* **257**, 88–94. 10.1016/j.expneurol.2014.04.019 (2014). 24792920 10.1016/j.expneurol.2014.04.019

[45] Gamal-Eltrabily, M., Martínez-Lorenzana, G. & González-Hernández, A. Condés-Lara, M. cortical modulation of nociception. *Neuroscience* **458**, 256–270. 10.1016/j.neuroscience.2021.01.001 (2021). 33465410 10.1016/j.neuroscience.2021.01.001

[46] Zhang, H. et al. The contribution of periaqueductal gray in the regulation of physiological and pathological behaviors. *Front. Neurosci.* **18**, 1380171. 10.3389/fnins.2024.1380171 (2024). 38650618 10.3389/fnins.2024.1380171 PMC11034386

[47] Keay, K. A., Clement, C. I., Owler, B., Depaulis, A. & Bandler, R. Convergence of deep somatic and visceral nociceptive information onto a discrete ventrolateral midbrain periaqueductal gray region. *Neuroscience* **61**, 727–732. 10.1016/0306-4522(94)90395-6 (1994). 7838371 10.1016/0306-4522(94)90395-6

[48] Assareh, N. et al. Bidirectional modulation of nociception by GlyT2^+^ neurons in the ventrolateral periaqueductal gray. *eNeuro* **10**, ENEURO0069–232023. 10.1523/ENEURO.0069-23.2023 (2023). 10.1523/ENEURO.0069-23.2023 PMC10270318 37253591

[49] Bagley, E. E. & Ingram, S. L. Endogenous opioid peptides in the descending pain modulatory circuit. *Neuropharmacology* **173**, 108131. 10.1016/j.neuropharm.2020.108131 (2020). 32422213 10.1016/j.neuropharm.2020.108131 PMC7313723

[50] Xie, L. et al. Divergent modulation of pain and anxiety by GABAergic neurons in the ventrolateral periaqueductal gray and dorsal raphe. *Neuropsychopharmacology* **48**, 1509–1519. 10.1038/s41386-022-01520-0 (2023). 36526697 10.1038/s41386-022-01520-0 PMC10425368

[51] Yin, J. B. et al. dmPFC-vlPAG projection neurons contribute to pain threshold maintenance and antianxiety behaviors. *J. Clin. Invest.* **130**, 6555–6570. 10.1172/JCI127607 (2020). 32841213 10.1172/JCI127607 PMC7685740

[52] Clement, C. I., Keay, K. A., Owler, B. K. & Bandler, R. Common patterns of increased and decreased fos expression in midbrain and pons evoked by noxious deep somatic and noxious visceral manipulations in the rat. *J. Comp. Neurol.* **366**, 495–515. https://doi.org/10.1002/(SICI)1096-9861(19960311)366:3<495::AID-CNE9>3.0.CO;2-%23 (1996). 8907361 10.1002/(SICI)1096-9861(19960311)366:3<495::AID-CNE9>3.0.CO;2-#

[53] Chen, H. et al. The functional and anatomical characterization of three spinal output pathways of the anterolateral tract. *Cell Rep.* **43**, 113829. 10.1016/j.celrep.2024.113829 (2024). 38421871 10.1016/j.celrep.2024.113829 PMC11025583

[54] Foreman, R. D., Schmidt, R. F. & Willis, W. D. Effects of mechanical and chemical stimulation of fine muscle afferents upon primate spinothalamic tract cells. *J. Physiol.* **286**, 215–231. 10.1113/jphysiol.1979.sp012615 (1979). 108391 10.1113/jphysiol.1979.sp012615 PMC1281567

[55] Treede, R. D., Kenshalo, D. R., Gracely, R. H. & Jones, A. K. The cortical representation of pain. *Pain* **79**, 105–111. 10.1016/s0304-3959(98)00184-5 (1999). 10068155 10.1016/s0304-3959(98)00184-5

[56] Rollman, G. B. & Lautenbacher, S. Sex differences in musculoskeletal pain. *Clin. J. Pain*. **17**, 20–24. 10.1097/00002508-200103000-00004 (2001). 11289085 10.1097/00002508-200103000-00004

[57] Wijnhoven, H. A., de Vet, H. C. & Picavet, H. S. Explaining sex differences in chronic musculoskeletal pain in a general population. *Pain* **124**, 158–166. 10.1016/j.pain.2006.04.012 (2006). 16716517 10.1016/j.pain.2006.04.012

[58] De Rui, M. et al. Pressure pain threshold of the cervico-facial muscles in healthy elderly people: The role of gender, age and dominance. *Gerodontology* **32**, 274–280. 10.1111/ger.12117 (2015). 26780382 10.1111/ger.12117

[59] Cairns, B. E., Hu, J. W., Arendt-Nielsen, L., Sessle, B. J. & Svensson, P. Sex-related differences in human pain and rat afferent discharge evoked by injection of glutamate into the masseter muscle. *J. Neurophysiol.* **86**, 782–791. 10.1152/jn.2001.86.2.782 (2001). 11495950 10.1152/jn.2001.86.2.782

[60] Nagakura, Y., Oe, T., Aoki, T. & Matsuoka, N. Biogenic amine depletion causes chronic muscular pain and tactile allodynia accompanied by depression: A putative animal model of fibromyalgia. *Pain* **146**, 26–33. 10.1016/j.pain.2009.05.024 (2009). 19646816 10.1016/j.pain.2009.05.024

