# Supplementary material for: Central projections of nociceptive input originating from the low back and limb muscle in rats
Source: Sci Rep. 2025 Jan 20;15:2552. doi: 10.1038/s41598-025-86832-z (PMC11747617; doi:10.1038/s41598-025-86832-z)
Supplement: Supplementary file 1 — Supplementary Material 1. [file 41598_2025_86832_MOESM1_ESM.docx]

**Supplementary Table 1. Statistical summary of Fig. 4**

| **Segment** | **Comparison** | **(A) Laminae**  **I−II** | **(B) Laminae**  **III−IV** | **(C) Laminae**  **V (−VI)** | **(D) Lamina**  **X** |
| --- | --- | --- | --- | --- | --- |
| **Th12** | MF/saline vs. MF/formalin | *p* = 0.9529 | *p* = 0.9146 | *p* = 0.8196 | *p* = 0.3053 |
|  | GS/saline vs. GS/formalin | *p* = 0.9986 | *p* = 0.3321 | *p* = 0.2523 | *p* = 0.3053 |
|  | MF/saline vs. GS/saline | *p* = 0.9967 | *p* = 0.8041 | *p* = 0.9968 | *p* = 0.8795 |
|  | MF/formalin vs. GS/formalin | *p* = 0.9649 | *p* = 0.9889 | *p* = 0.8594 | *p* = 0.8795 |
| **Th13** | MF/saline vs. MF/formalin | *p* = 0.7954 | *p* = 0.4288 | *p* = 0.8946 | *p* = 0.4525 |
|  | GS/saline vs. GS/formalin | *p* = 0.9386 | *p* = 0.1094 | *p* = 0.3532 | *p* = 0.8366 |
|  | MF/saline vs. GS/saline | *p* = 0.9998 | *p* = 0.9734 | *p* = 0.9993 | *p* = 0.9454 |
|  | MF/formalin vs. GS/formalin | *p* = 0.9749 | *p* = 0.8402 | *p* = 0.8401 | *p* = 0.6288 |
| **L1** | MF/saline vs. MF/formalin | *p* = 0.3298 | *p* = 0.4551 | *p* = 0.6010 | **p* = 0.0222 |
|  | GS/saline vs. GS/formalin | *p* = 0.7519 | *p* = 0.1145 | *p* = 0.7523 | *p* = 0.9834 |
|  | MF/saline vs. GS/saline | *p* = 0.9992 | *p* = 0.8858 | *p* > 0.9999 | *p* = 0.9764 |
|  | MF/formalin vs. GS/formalin | *p* = 0.8356 | *p* = 0.7803 | *p* = 0.9914 | *^#^p* = 0.0195 |
| **L2** | MF/saline vs. MF/formalin | **p* = 0.0216 | *p* = 0.5285 | *p* = 0.0530 | ****p* = 0.0003 |
|  | GS/saline vs. GS/formalin | *p* = 0.3434 | *p* = 0.4469 | *p* = 0.0860 | *p* = 0.9160 |
|  | MF/saline vs. GS/saline | *p* = 0.9978 | *p* > 0.9999 | *p* = 0.9945 | *p* = 0.9993 |
|  | MF/formalin vs. GS/formalin | *p* = 0.6899 | *p* = 0.9340 | *p* > 0.9999 | *^##^p* = 0.0043 |
| **L3** | MF/saline vs. MF/formalin | *****p* < 0.0001 | *p* = 0.5810 | ***p* = 0.0033 | *p* = 0.5102 |
|  | GS/saline vs. GS/formalin | *****p* < 0.0001 | *p* = 0.6998 | ****p* = 0.0008 | *p* = 0.9737 |
|  | MF/saline vs. GS/saline | *p* = 0.9986 | *p* = 0.7035 | *p* = 0.9968 | *p* = 0.9890 |
|  | MF/formalin vs. GS/formalin | *p* = 0.6899 | *p* = 0.4962 | *p* = 0.9945 | *p* = 0.9160 |
| **L4** | MF/saline vs. MF/formalin | *****p* < 0.0001 | *p* = 0.5620 | ****p* = 0.0002 | *p* = 0.2024 |
|  | GS/saline vs. GS/formalin | *****p* < 0.0001 | *p* = 0.1562 | *****p* < 0.0001 | *p* = 0.3539 |
|  | MF/saline vs. GS/saline | *p* = 0.9862 | *p* = 0.5585 | *p* = 0.7756 | *p* = 0.9709 |
|  | MF/formalin vs. GS/formalin | *p* = 0.1734 | *p* = 0.6440 | *p* = 0.9681 | *p* = 0.8659 |
| **L5** | MF/saline vs. MF/formalin | ***p* = 0.0013 | *p* = 0.5818 | *p* = 0.2178 | *p* = 0.0728 |
|  | GS/saline vs. GS/formalin | ***p* = 0.0015 | *p* = 0.4606 | *p* = 0.0860 | *p* = 0.9214 |
|  | MF/saline vs. GS/saline | *p* = 0.9953 | *p* = 0.4125 | *p* = 0.9984 | *p* > 0.9999 |
|  | MF/formalin vs. GS/formalin | *p* = 0.9916 | *p* > 0.9999 | *p* = 0.9914 | *p* = 0.2608 |
| **L6** | MF/saline vs. MF/formalin | *p* = 0.9998 | *p* = 0.9965 | *p* = 0.9993 | *p* = 0.9995 |
|  | GS/saline vs. GS/formalin | *p* = 0.9862 | *p* = 0.4312 | *p* = 0.2178 | *p* = 0.1639 |
|  | MF/saline vs. GS/saline | *p* = 0.9702 | *p* = 0.4116 | *p* = 0.7036 | *p* = 0.9905 |
|  | MF/formalin vs. GS/formalin | *p* > 0.9999 | *p* = 0.8653 | *p* = 0.7523 | *p* = 0.3373 |

Note: Statistical analysis was performed using two-way repeated measures ANOVA followed by Tukey’s multiple comparison test. **p* < 0.05, ***p* < 0.01, ****p* < 0.001, and *****p* < 0.0001, MF/formalin vs. MF/saline or GS/formalin vs. GS/saline. ^#^*p* < 0.05 and ^##^*p* < 0.01, MF/formalin vs. GS/formalin. **(A)** In laminae I−II: Significant effects of SEGMENT (F_7, 56_ = 11.89, *p* < 0.0001), MUSCLE (F_3, 8_ = 24.21, *p* = 0.0002), and SEGMENT × MUSCLE interaction (F_21, 56_ = 3.671, *p* < 0.0001). **(B)** In laminae III−IV: Significant effects of MUSCLE (F_3, 8_ = 7.957, *p* = 0.0087), but not SEGMENT (F_2.714, 21.72_ = 2.119, *p* = 0.1318) and SEGMENT × MUSCLE interaction (F_21, 56_ = 0.6113, *p* = 0.8929). **(C)** In laminae V (−VI): Significant effects of SEGMENT (F_7, 56_ = 6.489, *p* < 0.0001) and MUSCLE (F_3, 8_ = 28.74, *p* = 0.0001), but not SEGMENT × MUSCLE interaction (F_21, 56_ = 1.419, *p* = 0.1492). **(D)** In lamina X: Significant effect of MUSCLE (F_3, 8_ = 5.903, *p* = 0.0200), but not SEGMENT (F_7, 56_ = 0.679, *p* = 0.6891) or SEGMENT × MUSCLE interaction (F_21, 56_ = 1.477, *p* = 0.1242).
